# Protein drug target activation homogeneity in the face of intra-tumor heterogeneity: implications for precision medicine

**DOI:** 10.18632/oncotarget.14019

**Published:** 2016-12-15

**Authors:** Erika Maria Parasido, Alessandra Silvestri, Vincenzo Canzonieri, Claudio Belluco, Maria Grazia Diodoro, Massimo Milione, Flavia Melotti, Ruggero De Maria, Lance Liotta, Emanuel F. Petricoin, Mariaelena Pierobon

**Affiliations:** ^1^ Center for Applied Proteomics and Molecular Medicine, George Mason University, Manassas, VA, USA; ^2^ Department of Experimental Oncology, CRO-National Cancer Institute, Aviano, Italy; ^3^ Department of Pathology, CRO-National Cancer Institute, Aviano, Italy; ^4^ Department of Surgical Oncology, CRO-National Cancer Institute, Aviano, Italy; ^5^ Department of Pathology, Istituto Nazionale Tumori Regina Elena, Roma, Italy; ^6^ Department of Pathology, Fondazione IRCCS Istituto Nazionale Tumori, Milano, Italy; ^7^ Department of Pathology, Sacred Heart Catholic University of Rome, Roma, Italy

**Keywords:** intra-tumor heterogeneity, personalized therapy, kinase signaling, laser capture microdissection, reverse phase protein microarray

## Abstract

Introduction: Recent studies indicated tumors may be comprised of heterogeneous molecular subtypes and incongruent molecular portraits may emerge if different areas of the tumor are sampled. This study explored the impact of intra-tumoral heterogeneity in terms of activation/phosphorylation of FDA approved drug targets and downstream kinase substrates.

Material and methods: Two independent sets of liver metastases from colorectal cancer were used to evaluate protein kinase-driven signaling networks within different areas using laser capture microdissection and reverse phase protein array.

Results: Unsupervised hierarchical clustering analysis indicated that the signaling architecture and activation of the MAPK and AKT-mTOR pathways were consistently maintained within different regions of the same biopsy. Intra-patient variability of the MAPK and AKT-mTOR pathway were <1.06 fold change, while inter-patients variability reached fold change values of 5.01.

Conclusions: Protein pathway activation mapping of enriched tumor cells obtained from different regions of the same tumor indicated consistency and robustness independent of the region sampled. This suggests a dominant protein pathway network may be activated in a high percentage of the tumor cell population. Given the genomic intra-tumoral variability, our data suggest that protein/phosphoprotein signaling measurements should be integrated with genomic analysis for precision medicine based analysis.

## INTRODUCTION

The implementation of precision cancer therapy based on the underpinning individualized molecular profile of each tumor has become the new paradigm in oncology with a significant number of new precision drugs receiving approval from the US Food and Drug Administration (FDA) every year. This targeted approach has shown promising results especially when patients are allocated to different treatment options based on the molecular characteristics of the tumor [1–9]. Nearly all precision medicine clinical studies utilize core needle biopsies or fine needle aspirates as the preferred tissue collection method due to their relatively non/low-invasive nature compared to surgical excision. However, recent studies have indicated that tumors are characterized by significant inter- and intra-tumoral molecular heterogeneity. As a consequence, a different molecular architecture may emerge when different areas of the tumor are sampled or when primary tumors are compared to matched metastatic lesions [10–21]. Therefore, if tumors are indeed heterogeneous at the genomic clonal level, is a single biopsy sufficient to determine the most appropriate course of treatment for any given patient?

Because genomic instability is a characteristic of cancer cells, whether the heterogeneity observed is largely based on variability of “passenger” alterations (not causally significant for the tumorigenic process nor under selective pressure) and whether these alterations are a cause or consequence of the tumorigenic process still remains unclear [22]. While most of the recent studies exploring molecular heterogeneity have relied on genomic analyses, functional protein signaling events, largely driven by phosphorylation, are the actual drug targets of many of the new precision therapies (e.g. kinase/enzyme inhibitors). Consequently, the analysis of the complex protein-signaling network of tumor cells remains a central element for better understanding the impact that tumor heterogeneity has on the selection of treatment for cancer patients. For these reasons, it is critical to understand if protein-based data generated form different regions of the same sample are robust and consistent. Thus far relatively few studies have focused on the tumor molecular heterogeneity of the proteome and especially on the kinome/phosphoproteome, [12, 23–26]. Moreover, protein analysis of tumor tissue performed in the past under the auspices of tumor heterogeneity evaluation, have failed to utilize upfront cellular enrichment/purification techniques such as Laser Capture Microdissection (LCM). Such approaches are necessary to eliminate confounding analytical variability of different cellular populations (e.g. epithelial cells, endothelial cells, lymphocytes, fibroblasts, nervous structures etc.), along with the inability to predict the level of any given protein/phosphoprotein in a specific cell subpopulation [27].

A number of studies have already demonstrated that the molecular landscape of metastatic lesions significantly differ from primary tumors [13–21]. Indeed, systemic alterations in the phosphoprotein driven signaling architecture appear early in the tumorigenic process, such as in breast cancer and skin cancer studies [28,29]. However, the development of metastatic lesions is the main cause of cancer related-death, and most precision medicine trials are currently evaluating the molecular profile of the metastatic lesions directly to identify “actionable” alterations to be tailored by the new anti-cancer compounds. Moreover, due to its proximity in the vascular tree, the liver is one of the most common sites of metastasis for a variety of cancers such as CRC, breast, ovary, and it is one of most accessible visceral sites to biopsy with relatively low morbidity. With an eye towards applications in the context of precision medicine based molecular profiling and with these reasons as context, we sought to explore the nature of intra-tumoral variability in the context of protein/phosphoprotein signaling with a focus on “actionable” cell signaling pathways using hepatic metastasis from colorectal cancer (CRC) as a model for future, expanded analysis. Reverse Phase Protein Microarray (RPPA) coupled with LCM was used to assess the expression/activation level of selected targets for FDA approved compounds and downstream substrates across different regions of a metastatic lesion. Previous findings from our group have identified the AKT-mTOR pathway as highly activated in liver metastases from CRC compared to matched primary tumors along with a number of Receptor Tyrosine Kinases (RTKs) and the downstream substrate ERK [16]. For these reasons we further explored changes within the MAPK and the AKT-mTOR pathway across different areas of the same lesion.

## RESULTS

### Analysis of the signaling architecture of study set 1

**Figure 1:**
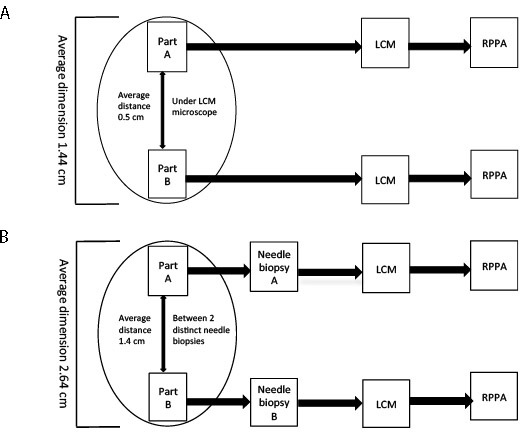
Sample collection and LCM-RPPA work flow For study set 1 two areas of the same specimen, separated by an average distance of 0.5 cm, were analyzed (**A**). For study set 2, two distinct areas, separated by an average of 1.4 cm, were harvested by a certified pathologist after surgical removal of the metastatic lesion and processed separately (**B**).

Study Set 1 included 6 surgical specimens with an average diameter of 2.5 cm. Only a portion of the original specimen (average of 1.4 cm) was used for this analysis. Tumor epithelial cells collected from two different areas of the same lesion were compared within and across patient(s). In all instances, the two areas analyzed (named “A” and “B” respectively) were separated by an average of 5 mm of tissue (Figure 1A). Unsupervised hierarchical clustering analysis was initially performed to evaluate the overall signaling architecture of two different areas of the same lesion.

**Figure 2:**
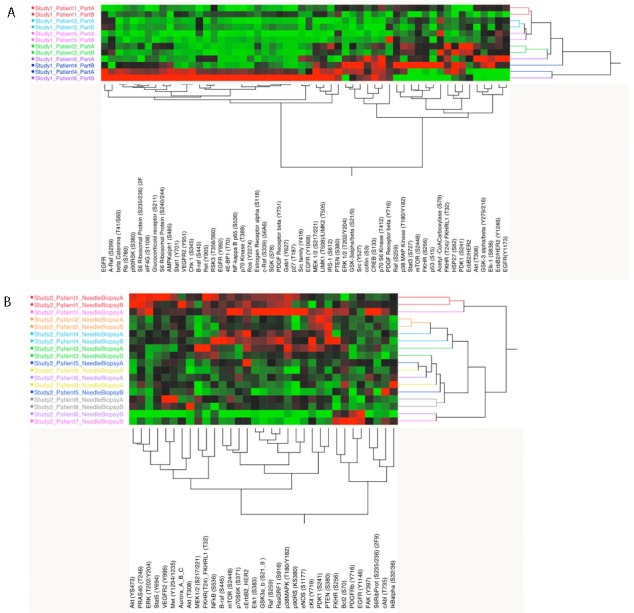
Unsupervised hierarchical clustering analysis for the full set of analytes measured for study set 1 and 2 (**A** and **B**). On the x-axis are listed the protein/phosphoprotein measured (56 and 33 respectively for study set 1 and 2). On the y-axis are reported the samples; same color was used for each pair of matched samples.

The 56 proteins/phosphoproteins measured in the first study set showed that in 4 of the 6 (67%) paired samples the overall signaling architecture was maintained across the different areas of the same tumor (Figure 2A). Because the data seemed to be driven mainly by the two samples with overall high signal (sample 6b and 4a), the unsupervised hierarchical clustering analysis was repeated after the two samples were removed; the second analysis was consistent with the original findings (Supplementary Figure 1).

**Figure 3:**
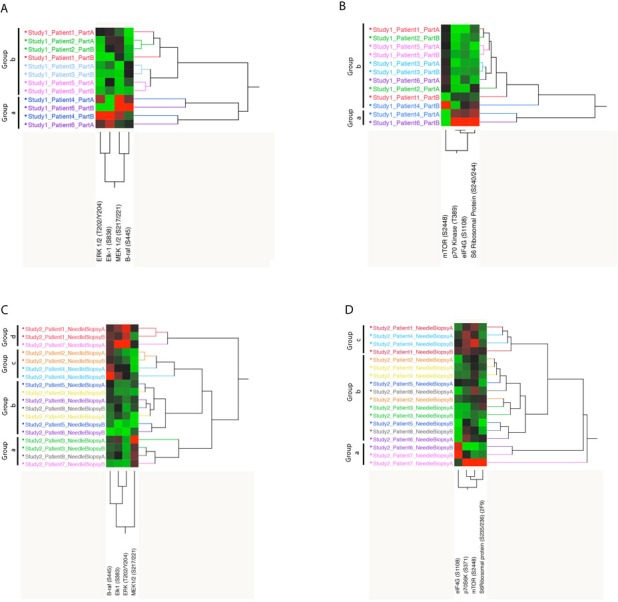
Unsupervised hierarchical clustering analysis Representation of the endpoints included in the MAPK and AKT-mTOR pathway modules for study set 1 (**A** and **B**) and 2 (**C** and **D**).

The analysis was then limited to proteins that are drug targets and downstream enzymatic substrates of either FDA approved targeted kinase inhibitors or experimental agents under investigation in clinical trials: the MAPK and the AKT-mTOR signaling pathways. As shown in Figure 3A and 3B, two distinct clusters were detected for both the MAPK and the AKT-mTOR pathway with one group of patients showing high activation of the druggable targets and substrates, and the second group presenting with low activation. Since high and/or low levels of drug target activation would likely be used to stratify patients to a targeted treatment, for each patient we evaluated how many times paired specimens were contained within the same cluster (e.g. group a and b). For the MAPK pathway all matched pairs were contained in the same group (Figure 3A), while for the AKT-mTOR pathway, 4 of the 6 matched paired (66.6%) where included in the same cluster (Figure 3B).

**Figure 4:**
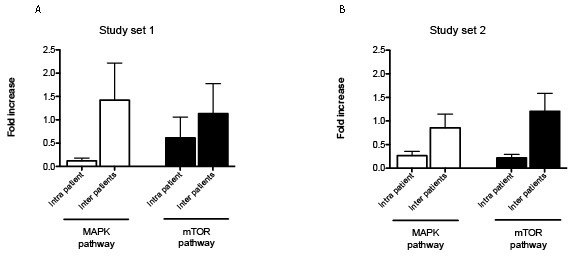
Representation of intra-tumor heterogeneity and heterogeneity across patients MAPK and mTOR pathway module were compared to assess the degree of heterogeneity within the same lesion (intra-patient variability) and across different patients (inter-patient variability) (**A** and **B** for study set 1 and 2 respectively). Fold change and standard error of the mean are shown for each protein.

To further investigate the role of tumor heterogeneity at the signaling level, fold changes between and within patients were evaluated for both pathways (Figure 4A). Fold changes for the MAPK pathway ranged between 0.04-0.73 within the same lesion and reached values of 5.06 when lesions from different patients were compared. Intra-patient fold change for the AKT-mTOR pathway was below 0.7 for 5 of the 6 cases, while it was between 0.01 and 3.57 across patients.

### Analysis of the signaling architecture of study set 2

Study Set 2 included metastatic lesions from 9 patients (Figure 1B). Immediately after surgical excision, two separate areas were isolated from each metastasis using 18-, 16- and 14- gauge needle. Samples were stored and processed independently. The average dimension of the metastases was 2.6 cm and the average distance between the two areas analyzed was 1.4 cm.

Unsupervised hierarchical clustering analysis of the activation/phosphorylation state of the 33 drug targets measured in study set 2 revealed that 5 of the 9 matched pairs (55.5%) were contained within the same cluster (Figure 2). As shown for study set 1, when the unsupervised analysis was limited to the MAPK and AKT-mTOR pathway, distinct clusters were detected based on the activation level of the different components of the module (Figure 3C and 3D). Four different clusters were identified for the MAPK pathway. The first cluster (group a) included 4 samples characterized by high activation of MEK. Of the four samples included in this group, two belonged to the same specimen. The second cluster (group b) showed overall low activation of the MAPK pathway. Of the seven samples included in this cluster, six belonged to matching pairs. The third cluster (group c) was characterized by increased activation of BRAF only; two matched pairs were grouped within this cluster. Finally the last cluster (group d) showed increased overall activation of the pathway. Of the three samples included in this group, two were collected from the same specimen.

Similarly, three major clusters were found for the AKT-mTOR pathway module. The first group (group a) showed an overall partial activation of the pathway. Of the three samples included in this first cluster, two belonged to the same patient. A large cluster with overall low activation of the pro-survival pathway included 11 samples (group b), 10 of which belonged to matching pairs. Finally group c showed high activation of the rapamycin target mTOR and direct downstream substrate p70S6K. A total of four samples collected from two patients were included in this cluster. Also in this case, intra-tumor heterogeneity in terms of fold change between matched pairs ranged between <0.01 and 1.06 for the MAPK pathway and between 0.03 and 1.04 for the AKT-mTOR pathway. Inter-tumor heterogeneity fluctuated between 0.13 and 2.62 for MAPK pathway and 0.45 and 3.76 for AKT-mTOR pathway (Figure 4B).

## DISCUSSION

The recent discussions concerning intra-tumoral heterogeneity and clonal expansion has opened a new debate on the validity of selecting targeted treatment(s) for cancer patients based on a single tissue biopsy [30]. Given the limitations of safe tissue collection for precision medicine trials, especially from patients with metastatic disease, it is important to understand biopsy-to-biopsy and intra-biopsy molecular variations. For example, we do not know whether the dominant activated signal pathways differ between tumor cells sampled at opposite ends of a core biopsy. If each tumor cell population has a characteristic dominant activated signal network that spans the whole population, then a single biopsy may be an accurate representation of the state of the whole heterogeneous tumor colony. On the other hand, if each part of the tumor is different, a set of biopsies would be required to collect a representative sample. In this study, we used an LCM-RPPA workflow to address this question with regard to the signaling architecture of metastatic lesions in the liver.

A number of studies have demonstrated that biopsies collected from different areas of the same lesion and across lesions have substantial variability at the genomic level with the intra-tumoral heterogeneity being much smaller then inter-tumoral heterogeneity [11–13,31–33]. Nonetheless, the impact of genomic heterogeneity on the proteome and its activation has not been deeply explored thus far [23–26,34,35]. An aspect of the current narrative concerning the intra-tumoral genomic heterogeneity observed in recent studies is whether a major contributor to the variability observed might be attributed to passenger genetic alterations that are not under selection pressure and thus vary more randomly across a given lesion. Because genomic studies may be unsuited for discriminating between drivers versus passenger alterations, we postulated that proteomic studies directly measuring the functional activation of the drug targets, themselves, may reveal new insight on the nature of inter- and intra-tumor heterogeneity.

The approach used in this study offers a number of attributes that distinguish it from previous work. First, the direct measurement of phosphorylated drug targets and downstream substrates provides a direct read-out on the functional signaling network of the tumor cells by providing information about which drug targets are truly activated and whether this activation is affecting the downstream members of the network. In vitro and in-/ex-vivo analyses have shown that the direct quantification of the activation level of targeted proteins and effectors can successfully be used to predict the therapeutic effect of biological compounds [36–41]. Second, our study uniquely used LCM for upfront tumor epithelium isolation and enrichment, an approach that has never been used before to explore tumor heterogeneity. Previous studies focused on inter- and intra-tumor variability analyzed the whole tissue lysate without upfront tumor epithelial cell isolation [23,25,26,35]. A number of publications have shown that upfront cellular enrichment is necessary when conducting functional proteomics studies because the different cellular component of the tumor microenvironment can deeply influence the accuracy of the results [27,42,43]. Without this upfront cellular enrichment, it is impossible to clearly determine whether any changes seen across a specimen are due to tumor epithelium molecular heterogeneity or whether they are simply caused by the differences in the amount of fibroblasts, adipocytes, leucocytes, endothelial cells, and nerve cells across the specimen. Since important signaling drug targets like AKT, mTOR, ERK are ubiquitously found in every cell type at various concentrations, it is impossible to control for this co-variable without isolating the subpopulation of cells of interest from the outset. Finally, the unique tissue collection approach we utilized for this study, coupled to upfront cellular enrichment via LCM, allowed us to control for a number of pre-analytical variables that are known to impact the integrity of the phosphoproteome and proteome (e.g. time between sample collection and storage, type of fixation method etc. and uncontrolled cellular heterogeneity in the tissue input) [44]. For example, because the two areas analyzed in study set 1 were isolated from the same cryosection via direct visualization under the microscope, this approach allowed us to control for variability in terms of collection time and storage across surgical specimens. The approach used for study set 2, on the other hand, allowed us to mimic sample collection under radiological guidance, a procedure that has become part of the standard of care of patients with metastatic disease. In addition, the two fragments analyzed were collected concomitantly after surgical excision of the metastatic lesion to limit variability attributable to collection time. Despite the unique and novel aspects of our study sets, our conclusions are constrained by small sample sizes similar to recent studies [23], and thus will require further validation in larger study sets in order to draw definitive conclusions

Overall, these pilot data indicate that the activation level of a large panel of drug targets and downstream substrates is quite reproducible/stable across different areas of the same metastatic lesion, especially compared to the heterogeneity seen across different patients. These data imply that for each metastatic colony a single dominant signaling pathway may drive a majority of the cellular population, regardless of presumed genetic clonal heterogeneity. This may signify the existence of dominant driving signaling pathway within the metastatic colony. Nonetheless, while the tumor cells throughout a single metastasis may be similar, they may still be different from other metastasis in the same or different organs. Drake and colleagues have recently measured the activation level of a small panel of kinases within and across different metastatic lesions from castration resistant prostate cancer [23]. In concordance with our data, the analysis showed that the phosphorylation levels were maintained across different lesions collected from the same patient even for metastases that developed at different organ sites [23]. As expected, a greater variation in the activated kinome was observed between lesions collected from different patients indicating that the inter-patient variability is much greater than the changes within a single individual. Similarly, Malinowsky and colleagues have evaluated tumor heterogeneity in primary breast cancer and matched metastatic lymph nodes. The results indicated that different areas of the primary tumors as well as the metastatic lesions were heterogeneous; nonetheless the differences seen within the lymph node lesions were slightly smaller than the one measured across different areas of the primary tumors. Interestingly, when tumor variability was evaluated within different patients, the coefficient of variations were almost double compared to one measured in the intra-tumor analysis. This study used whole tissue lysates obtained from formalin-fixed paraffin-embedded (FFPE) tissue sections and analyzed by RPPA [25]. It is well known that fixation in formalin of different areas of the tumor is time dependent, and the signaling network of large FFPE specimens can vary across the different areas of the lesions based on the time of fixation. Finally, by using whole tissue lysates where samples are not enriched for tumor epithelia, the signaling network identified by this study cannot be attributed to the cancer cells directly, but rather is an average of all the different cell subpopulations present in the tissue (including immune cells, nerves, fibroblasts etc). Finally, a recent work sampling the same metastatic lesion longitudinally during targeted treatment showed reproducible intra-tumoral signaling architecture of non- target molecules across different points in time [45]. Even if sample collection was carried out over time under CT guidance, and as, consequence, different areas of the lesion were sampled and analyzed, the data indicated that the signaling network was robust not only across areas of the same lesions, but at different points in time.

Our study has limitations that need to be addressed through further investigations. Due to the low number of samples enrolled in the study, our data need to be validated on an independent set where multiple areas of the same lesions and different lesions are collected from a larger cohort of patients. Finally, the study sets only included liver metastasis from colorectal cancer.

Despite the importance of liver metastasis in cancer progression, our analysis is obviously limited to a single organ site. The degree of heterogeneity in other metastatic sites such as bone, brain, chest wall, etc. are not addressed by our work. Moreover, while we interrogated the AKT-mTOR and MEK-ERK signaling pathways due to their high importance as druggable targets, the degree of intratumoral heterogeneity in signaling activation in other drug target networks remains to be more deeply explored. Nonetheless, our profiling of 55 kinase drug targets did indicate an overall stable signaling architecture.

Other types of cancers may have a different degree of intra-tumoral variability at the signaling level and it could be that the variabilityility is influenced by the microenvironment of the organ-sites. Matching genomic profiling was not available for the two study sets analyzed, and direct evaluation of the genomic variability needs to be further investigated, especially in relationship to the protein kinase driven signaling data. While we focused our tumor heterogeneity analysis on the activation/phosphorylation state of specific key signaling proteins, it is it is, unknown not know whether or not these protein pathways represent the functional/causal “drivers” of the tumor in any of the patients. Future studies that incorporate mulit-omic molecular analysis from multiple independent biopsies obtained from the metastatic lesion in the context of precision medicine studies with known clinical outcome will help to better define the impact of tissue heterogeneity.

This first protein-based drug target activation analysis of the tumor epithelium obtained from human metastatic lesions indicates that the activated signaling architecture is consistently observed within metastatic lesions collected from the same patient while each patient's tumor has distinct patient-specific signaling portraits. Incorporating proteomic and phosphoproteomic profiling into the precision medicine workflow is both possible and could be synergistic with genomic analysis [46]. If validated on an independent set of samples, these results could be critically important for precision medicine applications, especially in the context of tissue requirements for accurate assessment of the underpinning molecular detail.

## MATERIALS AND METHODS

Two independent study sets of liver metastases from CRC patients were evaluated in this analysis. Samples were obtained from the Centro di Riferimento Oncologico (Aviano, Italy), Istituto Regina Elena (Rome, Italy), and Istituto Nazionale Tumori (Milan, Italy) where sample collection was approved by the local IRB. Patients entered the study voluntarily and provided signed informed consent prior to sample collection. All samples were rapidly collected to minimize pre-analytical variables, snap-frozen, and stored at −80°C until the molecular analysis was performed.

### Laser capture microdissection

For each sample, 10 eight-micron cryo-sections were prepared. Tumor epithelial cells were isolated from the surrounding microenvironment using the ArcturusXT™ LCM System (Arcturus Bioscence, Mountain View, CA, USA). Approximately 10,000 to 15,000 epithelial cells were isolated from each sample on CapSure Macro LCM caps (Arcturus Bioscence, Mountain View, CA, USA). In brief, slides were first fixed in 70% ethanol, washed in deionized water, stained with hematoxylin (Sigma, St. Louis, MO, USA) and Scott's tap water substitute (Elec Micros Sci, Hatfield, PA, USA) followed by dehydration in 70%, 95%, 100% ethanol and xylene. To preserve post-translation modification such as phosphorylation, complete mini protease inhibitor tablets (Roche Applied Science, Indianapolis, IN, USA) were added to 70 % ethanol, deionized water, hematoxylin, and Scott's tap water substitute. Microdissected cells were lysed in 1:1 solution of Tris-Glycine SDS sample buffer (Life Technologies, Carlsbad, CA, USA) and Tissue Protein Extraction Reagent (T-PER Pierce, Rockford, IL, USA) with 2.5% β-mercaptoethanol (Sigma, St. Louis, MO, USA) [27]. Cell lysates were boiled for 8 minutes and stored at −80°C until further processed.

### Reverse phase protein microarray

#### Array construction

Using a 2470 Aushon arrayer equipped with 185 μm pins (Aushon BioSystems, Burlington, MA), cell lysates were printed in triplicate onto nitrocellulose-coated slides (Grace Bio-Lab, Bend, OR). For quality control purposes, standard curves were printed on each array along with the samples. To quantify the amount of total protein in each sample, selected arrays were stained with Sypro Ruby staining solution (Molecular Probes, Eugene, OR) following manufacturing recommendation [47].

### Immunostaining

Before proceeding with immunostaining, arrays were first incubated in Reblot Stripping solution (Chemicon, Temecula, CA) for 15 minutes, washed twice with PBS (Life Technologies, Carlsbad, CA) and incubated in I-Block (Tropix, Bedord, MA) for one hour. Using an automatic system (Dako Cytomation, Carpinteria, CA), arrays were then incubated with commercially available 3% hydrogen peroxidase solution, avidin-biotin blocking system, and protein block (Dako Cytomation, Carpinteria, CA). The expression/activation level of a panel of FDA approved and/or under investigation drug targets and their downstream effectors was measured using a single primary antibody targeting the protein and phosphorylation site of interest. Each antibody used on the array was previously validated using western blot to confirm its specificity [48]. Fifty-six and 33 primary antibodies were used for study set one and two, respectively (Supplementary Table 1). A commercially available tyramide-based avidin/biotin amplification system (CSA; Dako Cytomation, Carpinteria, CA) and fluorescent detection (LI-COR Biosciences, Lincoln, NE) were used to quantify the amount of protein present in each sample. Antibody stained slides were scanned using the Tecan laser scanner (Tecan PowerScanner Tecan group Ltd., Männedorf, Switzerland). Images were analyzed with MicroVigene 5.1.0.0 (Vigenetech, Carlisle, MA) as previously described [46]. Because the output of the RPPA platform is quantitative, the intensity values obtained from the analysis were reported on a continuous variable.

### Statistical analysis

Unsupervised hierarchical clustering analysis was performed using JMP version 11 (SAS Institute Inc., SAS, Cary, NC). The impact of tumor heterogeneity in two major pathways commonly targeted in different solid tumors was then evaluated. The MAPK pathway module of study set 1 and 2 included B-Raf (S445), MEK 1/2 (S217/221), Elk1 (S838) and Erk (T202/Y204). The AKT-mTOR pathway module included mTOR (S2448) and its downstream substrates p70S6K (T389), S6 Ribosomal Protein (S240/244) and eIF4G (S1108) in study set one and mTOR (S2448), p70S6K (S371), S6Ribosomal Protein (S235/236) and eIF4G (S1108) in study set two. Pathway activation module scores for each sample were generated by summing the single individual value for each endpoint included in a given module. For the assessment of the intra-tumor heterogeneity, fold changes of the pathway activation score within the two samples of the same patient were evaluated. Fold change differences across patients were calculated using the average values derived from each matched pair. Average values were then normalized on the lowest value of the series. Data were represented using bar graphs created with GraphPad version 5.0a (GraphPad Software, Inc., San Diego, CA).

## SUPPLEMENTARY MATERIAL FIGURE AND TABLE




