# Supplementary material for: Protein drug target activation homogeneity in the face of intra-tumor heterogeneity: implications for precision medicine
Source: Oncotarget. 2016 Dec 15;8(30):48534–44. doi: 10.18632/oncotarget.14019 (PMC5564706; doi:10.18632/oncotarget.14019)
Supplement: Supplementary file 1 [file oncotarget-08-48534-s001.pdf]

Protein drug target activation homogeneity in the face of intra-tumor heterogeneity: implications for precision medicine

Supplementary Material

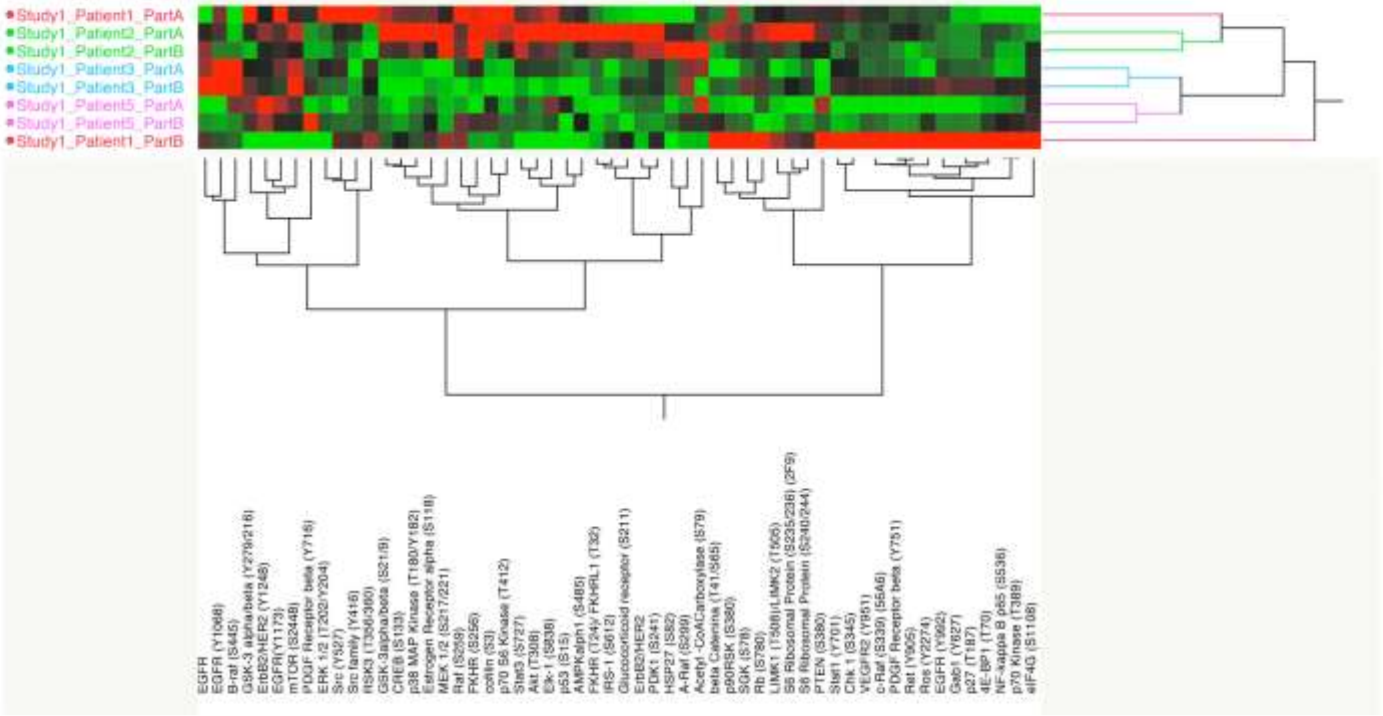

Supplementary Figure 1: Unsupervised hierarchical clustering analysis for samples included in study set 1 after two samples with overall high signaling activation were removed.

Supplementary Table 1: List of antibodies used for the analyses along with catalogue number and vendor.

For Supplementary Table 1 see in Supplementray Files.
